# Balloon Expandable Transcatheter Aortic Valve Implantation in Failing Self-Expandable Transcatheter Valve in Degenerated Surgical Bioprosthesis: Valve-in-Valve-in-Valve Implantation for the Treatment of Early Degenerative Prosthetic Insufficiency

**DOI:** 10.31486/toj.20.0011

**Published:** 2021

**Authors:** Ahmed Subahi, Rashid Alhusain, Rasikh Ajmal, Chaman Sohal, Omar E. Ali

**Affiliations:** ^1^ Department of Cardiology, Wayne State University/Detroit Medical Center, Detroit, MI; ^2^ Department of Internal Medicine, Wayne State University/Detroit Medical Center, Detroit, MI; ^3^ Cardiac Catheter Laboratory, Detroit Medical Center, Detroit, MI

**Keywords:** *Aortic valve*, *aortic valve disease*, *aortic valve stenosis*, *heart valve diseases*, *heart valve prosthesis implantation*, *transcatheter aortic valve replacement*

## Abstract

**Background:** Transcatheter aortic valve implantation (TAVI) has emerged as the standard of care for patients with severe aortic stenosis who are at high surgical risk. However, transcatheter valves can degenerate, and redo TAVI has been reported after surgical aortic valve implantation and post initial TAVI.

**Case Report:** We describe the case of a 70-year-old male who presented with decompensated heart failure secondary to severe prosthetic valve insufficiency. The patient had a history of distant triple coronary artery bypass surgery in 2004, surgical ascending aortic aneurysm repair and stentless aortic valve replacement in 2012, and transcatheter CoreValve (Medtronic) implantation in 2015 for the failing stentless aortic valve. In 2019, the patient presented with heart failure symptoms. A 29-mm SAPIEN 3 valve (Edwards Lifesciences) was implanted for the third time (valve-in-valve-in-valve) with excellent clinical and echocardiographic results and no evidence of coronary obstruction.

**Conclusion:** Early (<5 years) bioprosthetic valve insufficiency after initial valve-in-valve implantation can be successfully treated with a second TAVI.

## INTRODUCTION

Transcatheter aortic valve implantation (TAVI) has emerged as the standard of care for patients with severe aortic stenosis who are at high surgical risk,^1^ and in 2019, TAVI was validated for low-risk and young patients.^2^ Because of the use in young patients, the long-term durability of transcatheter valves has become relevant. Transcatheter valves can degenerate in a manner similar to other surgical bioprostheses, and redo TAVI has been reported after surgical aortic valve implantation as well as after initial TAVI.^3-6^ We present the case of a patient who presented with heart failure because of severe CoreValve (Medtronic) insufficiency and required a redo valve-in-valve (ViV) TAVI (third bioprosthetic valve).

## CASE REPORT

A 70-year-old male with hypertension, hyperlipidemia, and coronary artery disease with ischemic cardiomyopathy required triple coronary artery bypass surgery in 2004. Subsequently, the patient developed severe aortic stenosis and ascending aortic aneurysm, which necessitated surgical ascending aortic aneurysm repair and stentless aortic valve replacement in 2012. The patient had a 29-mm Freestyle modified inclusion root bioprosthesis (Medtronic) with 28-mm ascending aorta and hemiarch prosthesis (Vascutek Terumo Corp) placement. In 2015, the patient developed acute symptomatic aortic regurgitation after tearing the Freestyle bioprosthesis left cusp. He was deemed prohibitively high risk for conventional redo aortic valve replacement, so a ViV TAVI was performed using a 29-mm CoreValve. On discharge, the patient returned to his activities of daily living and reported feeling much improved with no cardiac symptoms on regular follow-up. Transthoracic echocardiogram (TTE) 6 months after the procedure showed a mean CoreValve gradient of 5.4 mmHg with no central or paravalvular leak.

**Figure 1.**
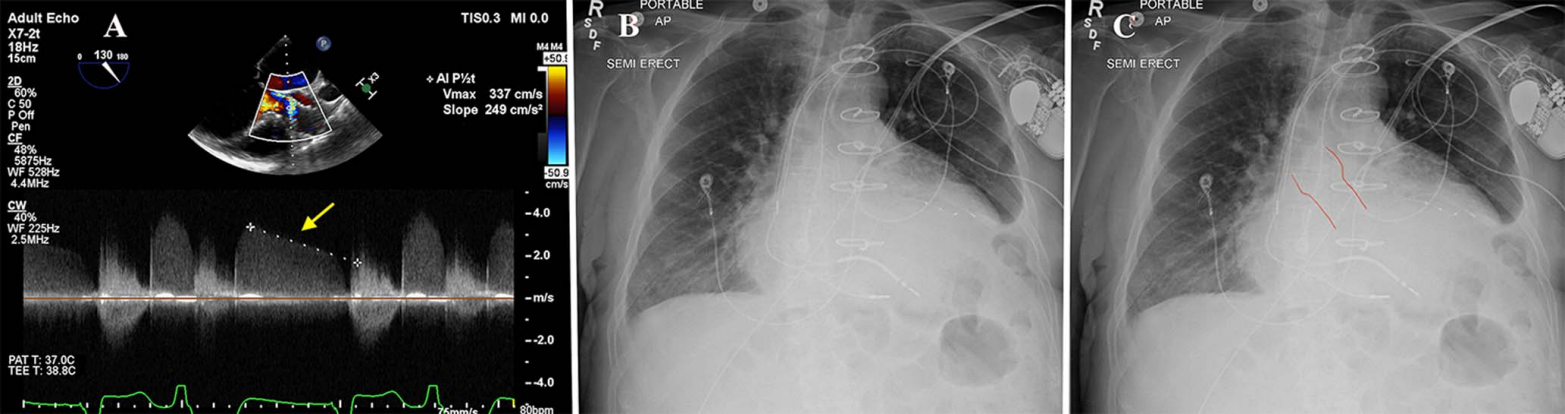
(A) Transesophageal Doppler with dense regurgitation signal and markedly reduced pressure half-time suggestive of severe CoreValve (Medtronic) insufficiency. (B) Anteroposterior chest x-ray before the CoreValve implantation, showing cardiomegaly and pulmonary congestion. (C) Anteroposterior chest-x-ray after the CoreValve implantation with the CoreValve outlined.

In 2019, the patient presented with acute New York Heart Association functional class IV heart failure symptoms. Chest auscultation was consistent with pulmonary edema. Biochemical testing revealed impaired renal function (urea 32 mg/dL, creatinine 1.26 mg/L, reduced estimated glomerular filtration rate 68 mL/min) and a markedly elevated brain natriuretic peptide level (3,294 pg/mL; reference range, <100 pg/mL). In transesophageal echocardiography (TEE), the CoreValve appeared well seated. Peak aortic systolic velocity was 184 cm/s. The CoreValve peak gradient was 13.5 mmHg, and the mean gradient was 6.4 mmHg. The left ventricular outflow to aortic valve velocity ratio (dimensionless index) was 0.55 (normal ratio, 0.28-0.55; <0.25 represents significant obstruction). Severe intraprosthetic regurgitation was seen. The regurgitant jet pressure half-time was 220 ms (severe regurgitation is <250 ms) (Figure 1). No periprosthetic regurgitation was appreciated. Holodiastolic flow reversal was observed in the descending aorta. Ejection fraction (EF) decreased to 35% with dilated cardiac chambers, secondary severe mitral regurgitation, and pulmonary hypertension.

The patient underwent a multidetector computed tomography (CT) scan to assess for a potential TAVI-in-TAVI procedure. The external iliac and common femoral arteries were suitable for transfemoral TAVI access. The structural heart team evaluated the aortic root CT to mitigate the anticipated complications, including ostial coronary occlusion with valve deployment. CT showed that the coronary ostia were high above the aortic annulus with ample room to accommodate the SAPIEN 3 valve (Edwards Lifesciences) leaflets. In addition, the sinus of Valsalva measured 29 mm with room to accommodate the SAPIEN 3 valve. The patient's clinical presentation and images were discussed at our structural heart disease team meeting. The patient was deemed prohibitively high risk for conventional redo aortic valve replacement (Society of Thoracic Surgeons predicted operative mortality [STS-PROM] score of 10.3%, Society of Thoracic Surgeons predicted operative morbidity and mortality [STS-PROMM] score of 46.75%) and was accepted for high-risk TAVI-in-TAVI.

**Figure 2.**
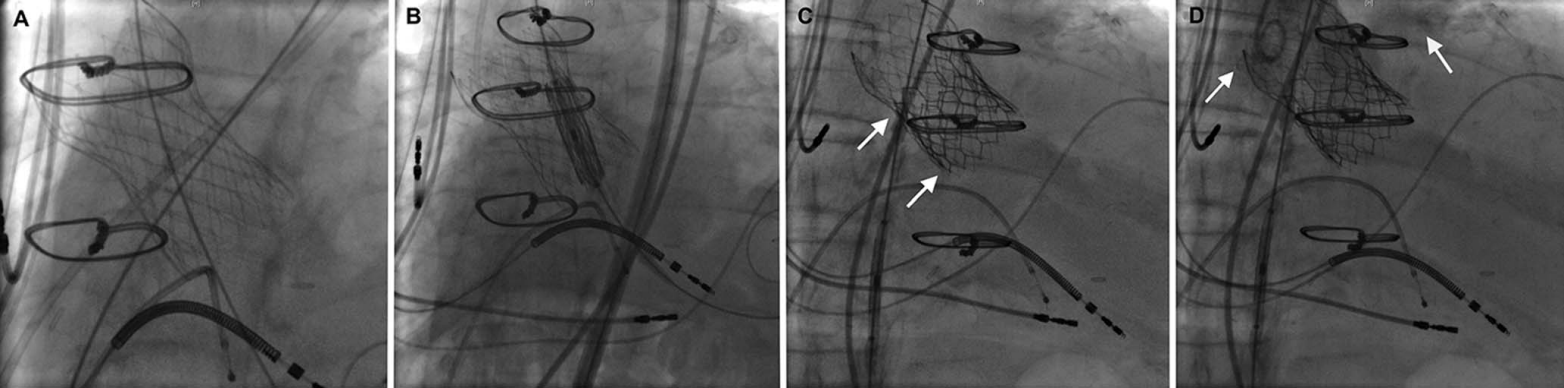
(A) Fluoroscopy before implantation shows the CoreValve (Medtronic). (B) Fluoroscopy at the time of implantation shows delivery of the SAPIEN 3 valve (Edwards Lifesciences). (C) Fluoroscopy shows the fully expanded SAPIEN 3 valve (arrows). (D) Fluoroscopy shows contrast injection in the aortic root after valve-in-valve-in-valve implantation (left arrow) with opacification of the left main artery (right arrow) and no contrast regurgitation into the left ventricle.

A 29-mm SAPIEN 3 valve was successfully deployed under fluoroscopic imaging with an immediate, significant reduction of the regurgitant flow (Figure 2). No evidence of a coronary obstruction or a paravalvular leak was seen on the check aortography. The patient was extubated from general anesthesia on the catheter laboratory table before being transferred to the ward. On postoperative day 4, the patient was discharged home. Prior to discharge, TTE showed no evidence of prosthetic aortic valve stenosis or regurgitation.

The patient was seen in the clinic 30 days after the redo TAVI. He was symptom-free and clinically euvolemic. TTE 30 days postimplantation showed an improvement of EF to 45%. Mean systolic aortic gradient was 3.4 mmHg, with no evidence of prosthetic aortic valve stenosis or regurgitation. The patient's severe mitral regurgitation regressed to mild-moderate, and the right ventricular systolic pressure decreased from 42 to 33 mmHg.

## DISCUSSION

The standardized definitions of structural valve degeneration by the European Association of Percutaneous Cardiovascular Intervention, the European Society of Cardiology, the European Association for Cardio-Thoracic Surgery, and the Valve-in-Valve International Data registry have resulted in increased uniformity in the evaluations of structural valve durability.^7^ In a 2018 study, the incidence of clinically relevant transcatheter valve degeneration after ViV TAVI for degenerated surgical bioprosthesis was 3% at up to 5-year follow-up. Meanwhile, the incidence of subclinical transcatheter valve degeneration was 15.1%.^8^ However, the long-term durability of ViV second or third TAVI for degenerated transcatheter bioprosthesis is unknown. Placement of a second or third TAVI occurs in 1.4% to 6.7% of patients, most often to salvage immediate complications of valve recoil, acute regurgitation, or perivalvular leak.^6^ However, a paucity of data is available regarding the feasibility, short-term outcomes, and long-term outcomes of double transcatheter ViV implantation in patients presenting with intraprosthetic insufficiency from transcatheter valve degeneration. Such patients may be unsuitable for emergency valve replacement surgery because of excessive risk of operative morbidity and mortality.

No published data are available on valve selection or sizing for TAVI inside an existing TAVI prosthesis.^6^ In TAVI degeneration, if a balloon-expandable valve is to be placed within a self-expandable valve, operators can use the original valve annulus measurements for selecting a valve size.^6^ Placement of a balloon-expandable valve inside a self-expandable valve offers more stability compared to a second self-expandable valve and can be an effective strategy to avoid perivalvular leak around the self-expandable valve.^6^ The radial force of the balloon-expandable valve is able to push the self-expandable valve frame out, and the skirt helps seal the annulus.^6^ Placement of a second self-expandable valve theoretically has a higher potential compared to a second balloon-expandable valve to make the coronary ostia more difficult to access, particularly if stent cells do not overlap. Coronary intervention after placement of the first TAVI is challenging but successful in more than 90% of cases.^6^ To our knowledge, no data are available on coronary access after TAVI-in-TAVI.^6^ Degeneration of the CoreValve within 4 years is unusual but has been described.^9^ The most important mechanism behind TAVI degeneration is leaflet degeneration.^7^ Leaflet degeneration occurs in a stepwise process, leading to tissue calcification and consequent valve stenosis or regurgitation.^7^

Several mechanisms of CoreValve leaflet degeneration pertinent to our patient could have been responsible for the severe regurgitation, including procedure-related factors, patient-related factors, and bioprosthesis-related factors. First, procedure-related factors include the trauma to the CoreValve leaflets that may occur when it is loaded into a delivery catheter before being established in its anatomic position.^7^ Second, the presence of turbulence in the aortic root (attributable to the anatomically abnormal root that has undergone the 2 previous surgeries of triple coronary artery bypass surgery and ascending aneurysm repair with stentless aortic valve replacement) and macrovascular atherosclerotic vasculopathy may affect blood flow, resulting in chronic mechanical stresses on the prosthetic valve leaflets, which then leads to early degeneration.^7^ Third, the preexisting degenerated bioprosthetic cusps, the configuration of the left ventricular outflow tract (usually noncircular), and the CoreValve oversizing can lead the self-expanding CoreValve to become noncircular (oval-shaped) after deployment, which could enhance degeneration.^7^ Other procedure-related factors that may have contributed to the bioprosthesis degeneration include leaflet endocarditis, thrombosis, and paravalvular leak. However, the previous procedure-related factors were less plausible compared to the preceding 3 factors after the structural heart team reviewed the patient's preprocedural laboratory workup, aortic root CT, TTE, and TEE. Patient-related factors in this case included dyslipidemia with atherosclerotic root aortopathy, chronic renal failure with abnormal phosphocalcic metabolism, and hypertension.^7^ Finally, bioprosthesis-related factors, including defects in bioprosthesis design, such as absence of antimineralization treatment or inaccurate valve dimensions leading to prosthesis-patient mismatch, may have also contributed to the early degeneration of the CoreValve.^7^

## CONCLUSION

Early (<5 years) transcatheter bioprosthetic valve insufficiency after initial ViV implantation can be successfully treated with a second TAVI.

## Notes

*This article meets the Accreditation Council for Graduate Medical Education and the American Board of Medical Specialties Maintenance of Certification competencies for Patient Care and Medical Knowledge.*
